# Deep Learning Based CAPTCHA Recognition Network with Grouping Strategy

**DOI:** 10.3390/s23239487

**Published:** 2023-11-29

**Authors:** Zaid Derea, Beiji Zou, Asma A. Al-Shargabi, Alaa Thobhani, Amr Abdussalam

**Affiliations:** 1 School of Computer Science and Engineering, Central South University, Changsha 410083, China; zabdulameer@uowasit.edu.iq (Z.D.); althobhanialaa@gmail.com (A.T.); 2 College of Computer Science and Information Technology, Wasit University, Wasit 52001, Iraq; 3 Department of Information Technology, Collage of Computer, Qassim University, Buraydah 51452, Saudi Arabia; as.alshargabi@qu.edu.sa; 4 Department of Computer Science, Collage of Computing and IT, University of Science &Technology, Sana’a 19065, Yemen; 5 Electronic Engineering and Information Science Department, University of Science and Technology of China, Hefei 230026, China; amr2010@mail.ustc.edu.cn

**Keywords:** computer vision, text-based CAPTCHA recognition, convolutional neural network, deep learning, text classification, image segmentation

## Abstract

Websites can improve their security and protect against harmful Internet attacks by incorporating CAPTCHA verification, which assists in distinguishing between human users and robots. Among the various types of CAPTCHA, the most prevalent variant involves text-based challenges that are intentionally designed to be easily understandable by humans while presenting a difficulty for machines or robots in recognizing them. Nevertheless, due to significant advancements in deep learning, constructing convolutional neural network (CNN)-based models that possess the capability of effectively recognizing text-based CAPTCHAs has become considerably simpler. In this regard, we present a CAPTCHA recognition method that entails creating multiple duplicates of the original CAPTCHA images and generating separate binary images that encode the exact locations of each group of CAPTCHA characters. These replicated images are subsequently fed into a well-trained CNN, one after another, for obtaining the final output characters. The model possesses a straightforward architecture with a relatively small storage in system, eliminating the need for CAPTCHA segmentation into individual characters. Following the training and testing of the suggested CNN model for CAPTCHA recognition, the experimental results demonstrate the model’s effectiveness in accurately recognizing CAPTCHA characters.

## 1. Introduction

The term CAPTCHA is an acronym that stands for “completely automated public Turing test to tell computers and humans apart”. Captchas were first proposed by Louis von Ahn et al. in 2003 [1] and have since become a common security feature in commercial applications to prevent malicious computer programs and bots. These tests are designed to be challenging for computers but easy for humans to solve. Captcha typically can appear in many forms [2] including text [3], image captcha [4], and sound CAPTCHA [5]. Researchers investigating CAPTCHA image recognition play an important role in identifying vulnerabilities and weaknesses in CAPTCHA systems. This research helps developers avoid these weak points in newly designed CAPTCHAs, improving internet security.

Despite being a favored and efficient technique to guard against harmful computer programs, the effectiveness of text-based CAPTCHAs can be enhanced by employing different methods. Some of these include introducing background noise, manipulating the text by warping, rotating, changing length, and combining characters. Nevertheless, with the progress in deep learning technologies, present defensive systems have lost their proficiency in dealing with CAPTCHA recognition systems. Hence, it is vital to create more intricate security measures to make text-based CAPTCHAs more robust against malicious assaults.

Deep learning technology [6,7,8,9] has greatly contributed to the widespread adoption of CAPTCHA recognition technology. The use of deep learning methods has shown remarkable effectiveness in identifying important features from input images and has a wide range of applications in various areas such as object detection [10,11,12] and image restoration [13,14,15]. This makes deep learning approaches a desirable alternative to creating strong algorithms that are capable of attacking text-based CAPTCHAs. While traditional digital image processing techniques are utilized in many CAPTCHA recognition algorithms, they still have limitations, such as insufficient ability for feature extraction and susceptibility to noise in input images. Consequently, these approaches are gradually replaced with more advanced deep learning techniques.

In most cases, text-based CAPTCHA recognition algorithms are categorized into two groups: those reliant on segmentation and those that operate without segmentation. Segmentation-based methods usually consist of two primary phases: the first is segmentation, where the CAPTCHA image is split into individual characters, and the second is character recognition, where these isolated characters are identified using a character recognition module. The segmentation step is crucial because it has a significant impact on the overall accuracy and efficiency of the system. However, many segmentation algorithms have limitations, resulting in reduced efficiency and effectiveness. As a result, researchers have begun exploring segmentation-free algorithms as an alternative to overcome the drawbacks associated with this process.

Presently, segmentation-free models have gained significant popularity in the field of CAPTCHA recognition. These models have the ability to directly recognize and categorize characters in CAPTCHAs without segmenting them into separate units. Furthermore, segmentation-free models regularly exhibit impressive levels of accuracy and efficiency. In contrast, algorithms that use deep learning to recognize CAPTCHAs rely on extensive datasets to effectively recognize the features of CAPTCHAs. Additionally, segmentation-free algorithms often require intricate architectures and demand substantial storage capacities, particularly while dealing with CAPTCHAs containing numerous characters.

This research recommends utilizing deep learning techniques, particularly convolutional neural networks (CNN), in implementing the recognition algorithm for CAPTCHAs. The recognition model, which is CNN-based, can efficiently perform the whole recognition process. The proposed CAPTCHA recognition algorithm involves making multiple copies of the input CAPTCHA image, generating unique binary images that represent the ordered location of each equal group of the CAPTCHA characters, and entering those copies sequentially into a well-trained CNN for character recognition. This algorithm eliminates the need for segmentation and enables direct and smooth recognition and classification of CAPTCHA input images using the attached binary images with a character grouping algorithm.

**Figure 1:**
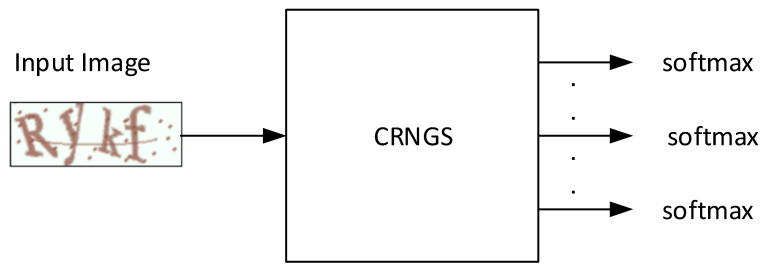
An illustration of the main operation of our proposed model. The input is a CAPTCHA image and the output is several softmax layers used to classify the CAPTCHA characters.

The proposed CAPTCHA recognition algorithm is given the name CAPTCHA Recognition Network with Grouping Strategy (CRNGS), and is depicted in Figure 1. This proposed model is a CNN with an adjustable number of softmax layers. The proposed grouping strategy divides the CAPTCHA characters into groups and enters them sequentially into the CNN. First, an arbitrary number of softmax layers is selected, less or equal to the number of CAPTCHA characters. This number of softmax layers divides the CAPTCHA characters into groups of characters’ groups so that the number of characters of each group (the group size) is equal to the number of softmax layers. Afterwards, we generate a number of binary images equals to the number of obtained groups. Then, we also make a number of copies of the input CAPTCHA image equal to the number of groups. Next, we attach each binary image to one CAPTCHA copy, and then these resultant CAPTCHA copies are sequentially entered into a CNN-based model that classifies the CAPTCHA characters group by group. By changing the number of softmax layers in our model, a new version of the model is implemented and used to recognize the CAPTCHA characters. Adjusting the number of softmax layers produces a range of versions of our model so that the version with the highest performance with a CAPTCHA scheme can be chosen as the final model.

In contrast to the majority of CAPTCHA recognition systems that do not use segmentation and have complicated structures, our suggested model has a straightforward, adaptable, and uncomplicated design. The model includes just one CNN, which adjusts the number of softmax layers and neurons in the output depending on the number of character classes. Additionally, the simplified design of our model significantly reduces its storage space, allowing it to remain small.

We have conducted our experiments on several CAPTCHA schemes datasets, including the BoC CAPTCHA scheme, Weibo CAPTCHA scheme, Captcha 0.3 CAPTCHA scheme, and Gregwar CAPTCHA scheme. The achieved results demonstrate that our proposed algorithm achieved an excellent performance with respect to the CAPTCHA recognition accuracy and the storage size on all adopted CAPTCHA schemes datasets.

The main contributions of this paper include:

- We present a novel CAPTCHA recognition algorithm that utilizes deep learning and binary images with character grouping, eliminating the requirement for segmenting CAPTCHA into individual characters.
- We implement a CAPTCHA recognition model with an adjustable number of softmax layers, leading to a range of versions of the model with selecting the model’s version with the highest performance.
- By implementing the character grouping algorithm, we have achieved a substantial reduction in the storage requirements of our model. Additionally, this has led to a simplification of the architecture within the CNN module, resulting in a more streamlined and efficient system.
- We conducted a series of experiments involving various CAPTCHA schemes datasets to evaluate the performance of our proposed CRNGS network. The results obtained from these experiments clearly demonstrate the high efficiency and effectiveness of our network in accurately recognizing CAPTCHA characters.

The upcoming parts of this document are arranged in this manner: In Section 2, various approaches and procedures for CAPTCHA identification and segmentation are introduced. The idea and details of the recommended CAPTCHA recognition algorithm are presented in Section 3. Section 4 discusses the datasets adopted and the configuration and parameters of the recognition Convolutional Neural Network (CNN), evaluates the accuracy of the proposed CAPTCHA recognition model, compares the outcomes, and provides a summary of the recommended CAPTCHA recognition algorithm. Finally, Section 5 concludes the study.

## 2. Related Work

Segmentation-based systems are widely relied upon for breaking Captcha. This approach views the segmentation step as a pivotal aspect of the recognition process. Various algorithms can be employed to segment text-based Captchas into individual characters. One notable study by Zhang et al. [16] utilized an improved vertical projection technique [17,18,19] for CAPTCHA segmentation. This technique effectively handled conglutinated characters by combining their sizes and locations within the projection histograms. The authors also addressed different types of conglutination segmentation. In a similar vein, Chellapilla and Simard [20] employed connected component models [21,22] to segment the Captcha schemes of Yahoo and Google. They achieved success rates ranging from 4.89% to 66.2%. However, it is computationally and time-consuming to perform multiple preprocessing operations when using vertical projection and connected component algorithms. Another approach by Hussain et al. [23] proposed a method for segmenting CAPTCHAs based on recognition. In this method, Artificial Neural Networks (ANNs) were trained to recognize manually cropped CAPTCHA characters. Subsequently, these trained ANNs were used to segment CAPTCHA images and crop their characters. However, it is important to note that this segmentation approach requires the application of ANNs to numerous extracted sub-windows to determine their confidence.

The efficiency of segmentation-based CAPTCHA recognition systems is heavily reliant on the character recognition module, which is responsible for the crucial task of identifying characters within the CAPTCHA. In a study conducted by Sakkatos et al. [24], they utilized template matching [25,26] as an approach to recognize characters by comparing them with predefined template characters. However, this method displayed a notable vulnerability when distinguishing characters that bore a striking resemblance to each other, which often resulted in errors. In contrast, Chen et al. introduced an innovative method known as “selective learning confusion class” (SLCC). This approach harnesses a two-stage deep convolutional neural network (DCNN) framework to accurately recognize CAPTCHA characters, especially those falling into the category of confusion classes. The SLCC method constructs subsets of confusion classes using a confusion relation matrix and a set partition algorithm. However, it is important to note a limitation of this approach, which is the significant increase in the storage size of the system due to the necessity of employing a new DCNN for each subset of confusion classes.

To address the limitations resulting from inefficient CAPTCHA segmentation systems, researchers have chosen deep-learning algorithms that recognize CAPTCHAs without segmentation, which directly recognize, and employed segmentation-free CAPTCHA recognition convolutional neural networks (CNNs) that recognize all CAPTCHA characters simultaneously without segmentation. They allocated specific neurons for each CAPTCHA character in the output layer to enable a classification process. While the model offers rapid recognition speed and eliminates the need for CAPTCHA segmentation, the storage size of the model increases as the CAPTCHA character count grows due to the expanding output layer neurons. Qing et al. presented another segmentation-free CNN model. In [27], the goal was to detect CAPTCHAs with connected and distorted characters. This model considered how adjacent characters are correlated to enhance recognition accuracy. However, the model employs different convolutional and fully connected layers on each character in the CAPTCHA, resulting in a complex architecture and an increase in the model’s storage size.

In their paper, Wang, Zhong, and Peibei Shi (2021) [28] present a novel approach for recognizing CAPTCHAs using a convolutional neural network (CNN) and focal loss. The study focuses on addressing the challenges associated with CAPTCHA recognition. However, this work has some limitations, such as the absence of comparative analysis with other established methods and the need for real-world testing to assess the effectiveness of their proposed approach in practical scenarios.

The authors in [29,30] utilized a segmentation-free model for CAPTCHA recognition, which combines a convolutional neural network (CNN) with an attention-based recurrent neural network (RNN). In this model, the CAPTCHA image is processed by the CNN to extract features and generate feature vectors. These vectors are then passed through a long short-term memory (LSTM) network, which converts them into a text sequence. This approach enables fast recognition and the ability to handle CAPTCHAs of varying lengths. However, due to the utilization of both CNN and RNN components, the architecture of this model is relatively complex, which may result in an increase in its storage size.

Ref. [31] presents an innovative method for cost-effective CAPTCHA breaking using open-source Python CAPTCHA libraries and deep learning technologies. It highlights the need for advanced AI defenses against CAPTCHA attacks. Ref. [32] focuses on evaluating the security of Hindi CAPTCHAs, as well as the analysis of colored CAPTCHAs. It employs various classifiers for segmentation and recognition, highlighting their performance. Ref. [33] introduced a novel CAPTCHA design incorporating the Hindi language, combining printed and handwritten characters to bolster security. Its distinctiveness is evident in its quest for a CAPTCHA with a “sweet spot” feature, harmonizing user-friendliness and machine-resistant characteristics. Ref. [34] introduces Style Matching CAPTCHA (SMC) employing neural-style transfer (NST) to combine security and user-friendliness. SMC tasks users with recognizing the style used in stylization, promoting a balanced approach to thwart bot attacks, including deep learning models.

Recently, Ref. [35] proposed a new method that uses convolutional neural networks with the attached binary images. This method has achieved high accuracy rates also in reducing the size of the system. The proposed method has some challenges with CAPTCHA recognition, including the need for large amounts of annotated data for training. Ref. [36] highlights the potential of combining deep learning and cognitive elements in CAPTCHA design. However, zxCAPTCHA includes an increased computational complexity and potential user learning curve, which could impact practical implementation. Ref. [37] introduces a novel approach for recognizing Persian CAPTCHAs using the TPS-Resnet-BiLSTM-ATTN deep learning model. Ref. [38] uses deep ensemble uncertainty estimation to detect and skip out-of-distribution CAPTCHAs, improving security by addressing a vulnerability in existing solvers. However, balancing security and usability by limiting image requests and wrong predictions may impact user experience, necessitating further research to strike the right balance.

In our proposed method, we introduce a generic method for CAPTCHA recognition where the CAPTCHA characters are organized into groups such that the group size is determined by the number of softmax layers and the number of groups determines the number of attached binary images required. As the number of softmax layers is changed, several versions of the model can be achieved for recognizing the characters of a CAPTCHA scheme.

## 3. Proposed Method

The following section describes our proposed algorithm. The CRNGS Approach is also explained. Next, we present the CAPTCHA recognition model’s internal structure.

### 3.1. Conceptualization of Proposed Recognition Method

**Figure 2:**
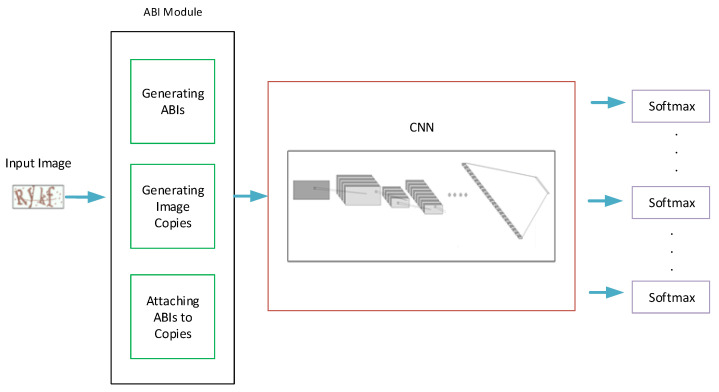
Framework description of the whole pipeline and structure of our proposed CRNGS CAPTCHA recognition model.

**Figure 3:**
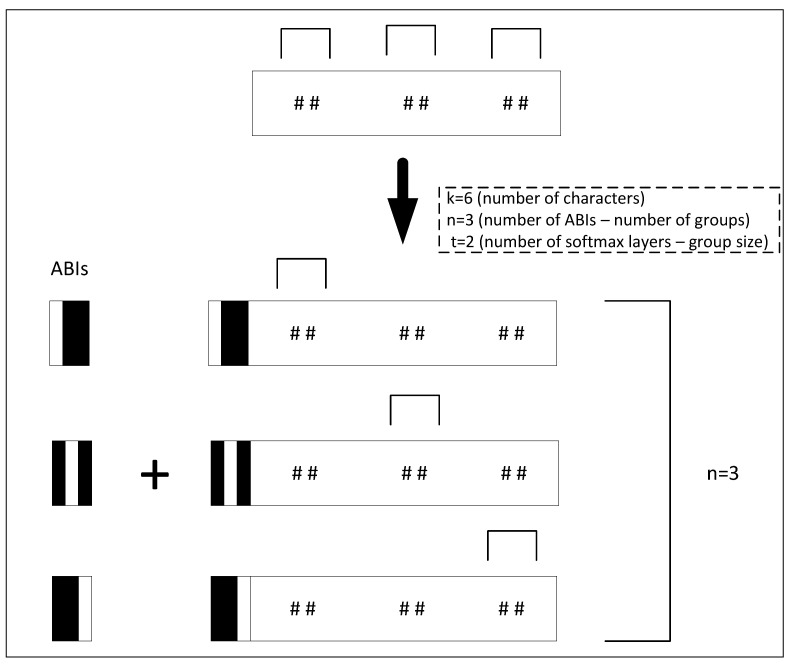
A description of the ABI module of our CRNGS model when the CAPTCHA image contains six characters (*k* = 6). The selected number of softmax layers *t* = 2, and the number of ABIs will be *n* = 3.

**Figure 4:**
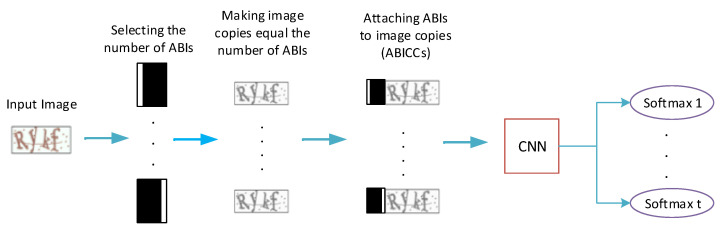
A general description of the pipeline of our proposed CRNGS model. For a CAPTCHA image with *k* characters, we first arbitrarily chose the number of softmax layers *t* and then we generate a number of ABIs *n* using Equation (1). After that, we generate *n* copies of the input image and attach the ABIs to the image copies to obtain *n* ABICCs. Thereafter, the ABICCs are entered sequentially into the CNN module and the characters are classified using the softmax layers.

The proposed CAPTCHA recognition model is mainly a CNN-based network that ends with softmax layers for character classification; Figure 2 provides an illustration for the whole model’s pipeline. It mainly consists of three parts: the attached binary images (ABI) module, the CNN, and the softmax layers. The ABI module is used to manipulate the input image to generate the suitable number of attached binary images (ABIs) and image copies. The CNN component is used to extract the features of the input images, while the softmax layers are used to classify characters. Figure 3 offers a detailed description for the ABI module, and Figure 4 illustrates the general structure of the proposed CAPTCHA recognition model.

For simplifying the explanation of the model’s operation, we need first to introduce some hyperparameters about the CAPTCHA image contents and the model’s structure. We will refer to the number of softmax layers as *t*, the number of ABIs as *n*, the number of characters in a CAPTCHA image as *k*, and the CAPTCHA characters’ categories as *m*.

In our proposed model, we start by selecting the number of softmax layers *t* in the output of our CAPTCHA recognition network. The number of softmax layers is chosen arbitrarily and it must be less than or equal the number of characters of the CAPTCHA *k*(t≤k). The number of softmax layers *t* is used to divide the *k* characters into groups, and each group is with size *t*. The number of groups is equal to the number of ABIs *n* and given by:

(1) $$n=\left\lceil \frac{k}{t}\right\rceil ,\;t\leq k$$

where ⌈⌉ is the popular ceiling function. As the number of ABIs *n* is determined, we generate *n* ABIs and *n* copies of the input CAPTCHA image.

**Figure 5:**
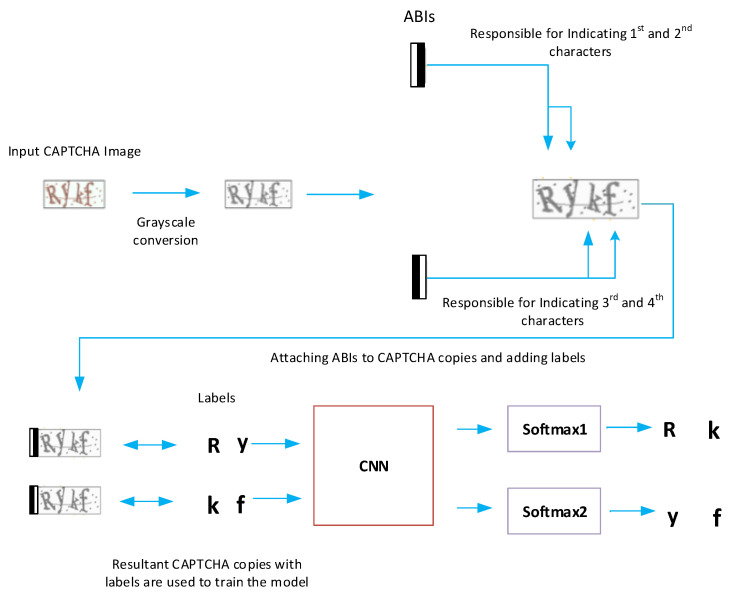
A description of the whole pipeline of our framework and the labeling process when the selected number of softmax layers (the group size) is set to *t* = 2 for a CAPTCHA image that contains *k* = 4 characters.

An ABI (Attached Binary Image) is a binary image (black and white image) that contains information that locates the character within a group. In our case, we choose to represent an ABI with a binary image with a black background and a vertical white bar. This white bar is first located on the left of the binary image for the first ABI and then gradually moved to the right for the remaining ABIs until it reaches the right edge of the binary image for the last ABI, as shown in Figure 3. The ABIs are used to divide the *k* characters of the CAPTCHA input image into *n* groups. The 1st ABI will be responsible for locating and recognizing the 1st group of characters, the 2nd ABI will be responsible for the 2nd group of characters, and so on. Within each of the *n* groups of characters, the 1st softmax layer is responsible for recognizing the 1st character, the 2nd softmax layer is responsible for the 2nd character, and so on. After that, each ABI is attached to a copy of the input image to generate a new image called Attached Binary Image with CAPTCHA copy (ABICC). Now, we end up with *n* ABICC copies which are then fed one after the other into the CNN network. Each of the *n* ABICCs is responsible for locating one of the *n* groups, and the *t* softmax layers are utilized to recognize the *t* characters of the group. This way, all the *k* characters of the CAPTCHA image can be recognized. Since the ABIs within the ABICCs include information about the location of the groups of characters and the softmax layers locate and recognize the characters within the groups, the CNN network can be trained to locate and recognize all characters of the CAPTCHA image. Figure 5 provides an illustration for the CAPTCHA and grouping strategy when the number of CAPTCHA characters *k* = 4 and the number of selected softmax layer *t* = 2.

### 3.2. Procedure of Proposed CAPTCHAs’ Recognition Algorithm

The fundamental procedure of the proposed CAPTCHAs’ recognition algorithm can be described with the following steps:

1. First, for a CAPTCHA with *k* characters, we arbitrarily determine the number of softmax layers *t* (t≤k).
2. We generate *n* copies of the CAPTCHAs input image according to Equation (1).
3. We define *n* binary images (ABIs) so that each binary image is given a unique number between 1 and *n*. For instance, the first binary image is given the number 1 and the second binary image is given the number 2, and so on.
4. Each binary image (ABI) is attached to one copy of the *n* copies of the CAPTCHA, ending up with *n* ABICCs.
5. For training the proposed CAPTCHA recognition algorithm, labels are added to each ABICC so that if we assume *t* = 2, then the ABICC with an ABI of number 1 is given the first characters’ group (first and second characters of CAPTCHA) to be its label, and ABICC with an ABI of number 2 is given the second character’s group (third and fourth characters of CAPTCHA) to be its label and so on.
6. Those ABICCs with their labels are used to train a powerful CNN to classify and recognize CAPTCHA characters. If we assume *t* = 2, then the ABI of an ABICC represents two character’s order or location and the label represents the two characters classes. This CNN classifies the input CAPTCHA copy characters into different *m* classes.
7. The ABICCs will be recognized using this well-trained CNN serially one by one using the softmax layers such that every softmax layer classifies one character and the desired output (CAPTCHA individual characters) will be achieved. Figure 5 shows the whole procedure of proposed CAPTCHA recognition algorithm when the number of softmax layers *t* is set to 2.

### 3.3. Structure of the Proposed CRNGS Approach

After preparing the *n* ABICCs, these images are entered into our proposed CAPTCHA recognition model in sequence to recognize the output characters. Our network is composed of CNN and softmax layers. The CNN is used to encode input ABICC and extract its visual features. The architecture of the CNN can be a popular architecture such as VGG, ResNet, and so on, or any other private CNN architecture. Then, the extracted visual features are entered into the softmax layers to classify the CAPTCHA characters.

Let *I* refer to the input image and *A* refer to the ABI. Then, the ABICC can be obtained by:

(2) C=[I;A]

where *C* refers to the ABICC and [ ] is the concatenation operation. Then, the ABICC is entered into the CNN for extracting features and classifying characters. The ABICC extracted features are given by:

(3) E=CNN(C)

Then, these extracted features are delivered to the softmax layers which are utilized to classify the characters, as follows:

(4) $$D_{i}=W_{i}\cdot E,\;i\in \left[1,2,\ldots ,t\right]$$

(5) $$p_{i}=softmax\left(D_{i}\right),\;i\in \left[1,2,\ldots ,t\right]$$

where $p_{i}\in R^{m}$ refers to the i−th probability distribution over all character categories. $W_{i}$ refers to the i−th trainable weights matrix. The softmax function is performed on the $D_{i}\in R^{m}$ vector and is defined as:

(6) $$D_{i}=\left(d_{i1},d_{i2},\ldots ,d_{im}\right),\;i\in \left[1,2,\ldots ,t\right]$$

(7) $$softmax\left(d_{il}\right)=\frac{e^{d_{il}}}{\sum_{q=1}^{m}e^{d_{iq}}},\;l\in \left[1,2,\ldots ,m\right]$$

During training the proposed CAPTCHA recognition network, we adopted the cross-entropy loss function. The loss for each softmax layer $loss_{i}$ is given by:

(8) $$loss_{i}=-\log\left(p_{i}\right),\;i\in \left[1,2,\ldots ,t\right]$$

and the total loss function $loss_{T}$ is defined as:

(9) $$loss_{T}=\sum_{i=1}^{t}loss_{i}=\sum_{i=1}^{t}-\log\left(p_{i}\right)$$

The proposed CAPTCHA recognition approach encompasses several versions of the model. Since *t* can be chosen arbitrarily to be any number between 1 and *k* inclusive, we can achieve *k* versions of the proposed model. In fact, we can implement all the *k* versions of the model and train them with the CAPTCHA images and then decide the most appropriate model in terms of accuracy, complexity, storage size, and training time. When t=k, then we achieve a multi-label classification model in which the ABIs are no longer needed. Conversely, when *t* = 1, we will obtain the maximum number of ABIs, which is *k*, so that each single character will have its corresponding ABI. However, when *t* is chosen to be any other number between 1 and *k* exclusive, a (*K*-2) versions of the model can be implemented with the grouping strategy, and the opportunity for obtaining higher recognition accuracy will be increased. It is worth mentioning that the introduced grouping strategy is feasible as long as the CAPTCHA scheme has a fixed number of characters.

## 4. Experiments and Results

In this section, we provide information about four datasets that were employed to train, validate, and test the CRNGS model, along with an explanation of how the labeling process was carried out. Thereafter, the CNN structure and the training parameters of the CRNGS model are presented. Following that, the accuracy of the model is evaluated. We then present the outcomes of a comparison between the CRNGS algorithm and other CAPTCHA recognition systems, and discuss the strengths and weaknesses of the CRNGS algorithm.

### 4.1. Used Dataset and Labeling Description

**Figure 6:**
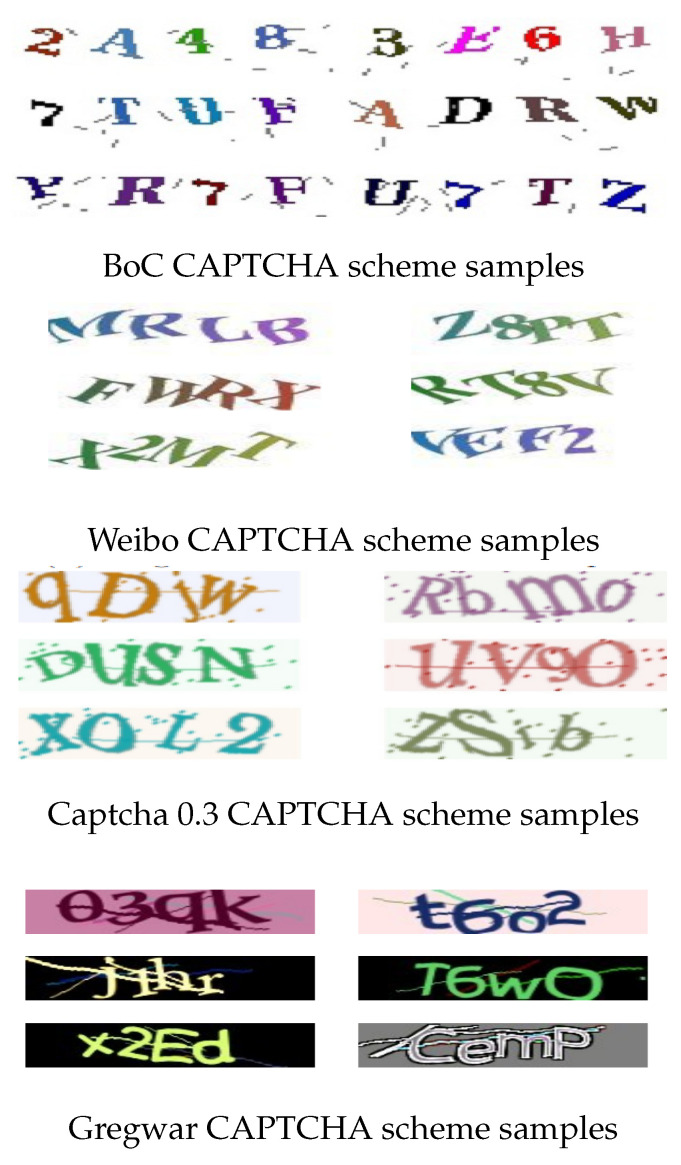
Samples of CAPTCHA image datasets adopted in our CRNGS model.

To perform CAPTCHA recognition tasks, we acquire CAPTCHA images either by gathering them from live online platforms or by utilizing CAPTCHA generation software. To implement our experiments, we employ four different schemes for obtaining CAPTCHA datasets: Bank of China (BoC) (https://ebsnew.boc.cn/boc15/login.html/ (accessed on 8 October 2023)), Weibo (https://www.weibo.com/) (accessed on 8 October 2023), Captcha 0.3 and Gregwar. Figure 6 shows samples of the four CAPTCHA schemes.

#### 4.1.1. Bank of China (BoC) Captcha Scheme

The Bank of China, which is among the four biggest government-owned commercial banks in China, has a vast presence in over 50 countries with over 10,000 branches both domestically and internationally. To prevent automated programs from exploiting its online services, the bank employs countermeasures such as distortion, character overlapping, rotation, and warping in its CAPTCHA scheme, which includes four characters belonging to either uppercase English letters or numeral digits. As a dataset, we have gathered, classified, and labeled a total of 70,000 random CAPTCHA images from the Bank of China manually. The CAPTCHAs employed by BoC consist of four characters, which can be either numerals or uppercase English letters. However, certain characters are not included in these CAPTCHAs, namely 0, 1, 5, C, G, I, L, O, Q, S.

#### 4.1.2. Weibo CAPTCHA Scheme

Weibo, a highly-ranked website by Alexa and one of the world’s most popular websites, stands as one of China’s largest social media platforms. As of 2022, the company boasted an impressive monthly active user base of 586 million individuals. Weibo has implemented a CAPTCHA system fortified with various resistance mechanisms, including distortion, character overlapping, rotation, and warping. These CAPTCHAs typically comprise four characters, which can be either numeral digits or uppercase English letters. However, certain characters such as 0, 1, 5, D, G, I, Q, and U are deliberately excluded from the character set used in these CAPTCHAs. To advance our research in CAPTCHA recognition, we compiled a comprehensive dataset. This dataset was painstakingly curated through manual collection and labeling efforts, encompassing a total of 70,000 randomly selected Weibo CAPTCHA images. This dataset serves as a valuable resource for further studies and experimentation in the field of CAPTCHA recognition and security.

#### 4.1.3. Captcha 0.3 CAPTCHA Scheme

Captcha 0.3 is a Python library that generates CAPTCHAs. It is free and open-source. Our choice for CAPTCHA images includes four characters, which can be either numeric values from 0 to 9, uppercase English letters from A to Z, or lowercase English letters from a to z. We have created a dataset of 70,000 CAPTCHA images. Each CAPTCHA image was generated randomly, ensuring that there are no repeated or duplicated images within the dataset. To enhance the security of the CAPTCHAs, we have added lines passing through the characters and included noisy dots in the background. The font “liberbaskerville-regular” was selected for generating these CAPTCHA images. Figure 6 provides some samples of the CAPTCHA scheme images used in our dataset.

#### 4.1.4. Gregwar CAPTCHA Scheme

The Gregwar PHP library excels at generating CAPTCHAs designed to thwart bot intrusions effectively. It employs a range of security features, including noise lines, colored backgrounds, and rotation, enhancing CAPTCHA robustness. These CAPTCHAs thoughtfully incorporate four characters drawn from three character classes: numerals, uppercase letters, and lowercase letters. In support of research in CAPTCHA recognition and cybersecurity, we meticulously constructed a dataset encompassing a substantial 70,000 Gregwar CAPTCHA images. Throughout the dataset creation process, the utmost care was taken to ensure that no duplicate characters or repetitions existed within each CAPTCHA, reinforcing its quality and integrity. This dataset serves as a valuable resource for researchers, providing a solid foundation for in-depth exploration and experimentation within the realm of CAPTCHA recognition and digital security.

#### 4.1.5. Preparing Dataset Images and Labeling Description

Throughout this study, the proposed model’s versions with their specific number of softmax layers will be denoted as CRNGS_*x*s, where *x* refers to the number of softmax layers. For example, CRNGS_2s refers to model’s version with 2 softmax layers. To simplify the CAPTCHA dataset scheme, we divided it into three sets: training, testing, and validating sets. Each set consists of a specific number of CAPTCHA images. The selection of images for training, testing, and validating sets is conducted randomly to avoid any biases.

For using the CAPTCHA schemes images to train and test our proposed models, we need first to perform some preprocessing steps on the CAPTCHA images and prepare them to be appropriately utilized in our models. First, all CAPTCHA images are converted to grayscale and reshaped to a size of 96 × 280 pixels. Then, for a CAPTCHA image that consists of *k* characters, we first arbitrarily select *t* softmax layers for the CNN and calculate the number of ABIs *n* according to Equation (1). Then, we generate *n* ABIs of size 96 × 40. Next, we create *n* copies of the CAPTCHA image. Thereafter, we attach each of the *n* ABIs to one of the *n* CAPTCHA image copies resulting in *n* ABICCs with size of 96 × 320. These ABICCs along with their corresponding labels will be directly used to train the CAPTCHA recognition network. Since we will generate *n* ABICCs for each CAPTCHA image, then we will end up with a new dataset that is *n* times the original CAPTCHA image dataset. In other words, if the CAPTCHA scheme dataset is originally composed of $\bar{Z}$ CAPTCHA images, and the selected number of ABIs is *n*, then the new dataset that will be used to train and test our network will be composed of (*n*×$\bar{Z}$) ABICCs images.

As an illustration, consider the CRNGS_2s model, where the number of softmax layers *t* is 2, the number of CAPTCHA characters *k* is 4, and the number of ABICCs according to Equation (1) *n* is 2, and every generated copy of a CAPTCHA is labeled using a two-character label. This labeling procedure involves allocating distinct character labels to each copy based on the original CAPTCHA image’s four-character text. Specifically, the first copy of the CRNGS_2s CAPTCHA is assigned the first and second characters from the original four-character text, while the subsequent copy is assigned the third and fourth characters. Following this labeling process, distinct dataset structures emerge, yielding a total of 140,000 images (2 copies × 70,000 CAPTCHA images) for each resulting dataset set, each accompanied by its respective character labels. These resultant dataset configurations are then utilized separately for training, validating, and testing our CRNGS model. The training set encompasses 100,000 images (2 copies × 50,000 CAPTCHA images), the testing set is constituted of 20,000 images (2 copies × 10,000 CAPTCHA images), and the validation set comprises another 20,000 images (2 copies × 10,000 CAPTCHA images).

The same process is followed for “CRNGS_3s” where the number of CAPTCHA characters *k* is 4, the number of softmax layer is *t* = 3, and the number of copies (groups) according to Equation (1) is *n* = 2. For the CRNGS_1s model, the number of softmax layer is *t* = 1, and the number of copies (groups) according to Equation (1) is *n* = 4. The training set is composed of 200,000 images (4 copies × 50,000 CAPTCHA images), while the testing set encompasses 40,000 images (4 copies × 10,000 CAPTCHA images). Similarly, the validation set consists of 40,000 images (4 copies × 10,000 CAPTCHA images). In the case of the CRNGS_4s model, the softmax layer count is denoted by *t* = 4, and the number of copies is represented by *n* = 1. Specifically, the training set includes 70,000 images (1 copy × 50,000 CAPTCHA images), the testing set is comprised of 10,000 images (1 copy × 10,000 CAPTCHA images), and the validation set involves 10,000 images (1 copy × 10,000 CAPTCHA images).

In our case, for generating the ABIs, we chose to represent an ABI with a binary image of size (96 × 40) so that the background of the image is black with a vertical white bar. The vertical bar starts to the left of the image for the first ABI, and it keeps moving to the right for the remaining ABIs until it reaches the right edge of the image for the last ABI. The generation of the ABIs and attaching them to the CAPTCHA images copies can be easily programmed in a preprocessing step, and the ABIs in our method are indeed generated automatically before the training and testing phases for each CAPTCHA scheme. Figure 3 provides an illustration for the ABIs for *n* = 3.

### 4.2. Structure and Parameters of Proposed CRNGS Model

**Figure 7:**
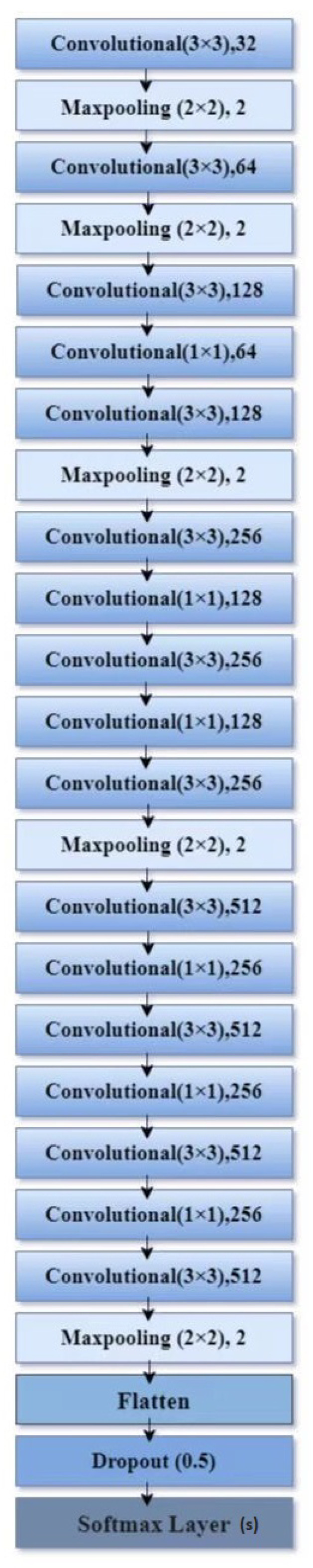
The architecture of the CNN adopted in our proposed model in terms of Convolutional, maxpooling, flattened, and softmax layers.

The proposed CRNGS model is inspired by the model-5 architecture introduced in a reference paper [39]. It consists of 17 convolutional layers, 5 maxpooling layers, 1 flattened layer, 1 dropout layer, and softmax layers. In order to enhance the training process and improve feature extraction, the parameters of the CRNGS have been adjusted. Figure 7 illustrates the complete architecture of the CRNGS model. The softmax layer at the output contains neurons corresponding to the number of character classes used in the CAPTCHA scheme. For instance, if the CAPTCHA scheme employs 62 character classes (including 10 numeral digits, 26 uppercase English letters, and 26 lowercase English letters), the softmax layer of the CRNGS model will consist of 62 neurons, with each neuron representing a specific character class. To quantify the differences between predicted and true classes during training, the cross-entropy loss function is employed for the proposed CAPTCHA recognition, CNN. After experimentation, a learning rate of 0.00001 has been determined as optimal for optimizing the loss function using the Adam optimizer. The training process involves performing 120 epochs.

### 4.3. Accuracies of Proposed Models

The individual characters’ accuracies as well as the total character accuracy and overall CAPTCHA accuracy of the CRNGS_1s, CRNGS_2s, CRNGS_3s, and CRNGS_4s models on the BoC, Weibo, 0.3 Captcha, and Gregwar CAPTCHA schemes are shown in Table 1, Table 2, Table 3 and Table 4, respectively. For the BoC CAPTCHA scheme shown in Table 1, it can be observed that the CRNGS_4s model achieved the highest performance in all accuracies. For the Weibo CAPTCHA scheme in Table 2, the CRNGS_1s model attained the highest score in almost all accuracies except the third character accuracy. For the 0.3 CAPTCHA scheme results listed in Table 3, the CRNGS_4s attained the highest results in terms of the total character accuracy and overall CAPTCHA accuracy.

For the Gregwar CAPTCHA scheme in Table 4, the CRNGS_1s exceeds the other models with respect to most of the accuracies except the third character accuracy. It can be noticed from the attained results that one model version achieves high performance for one CAPTCHA scheme, while it attains lower scores for another scheme. As a result, for achieving the best result for a CAPTCHA scheme, we can train different versions of the model with a different number of softmax layers and then select the model version with the highest performance.

### 4.4. Comparison Results

The comparison results between our proposed models and other modes in terms of BoC, Weibo, Captcha 0.3, and Gregwar CAPTCHA schemes are listed in Table 5, Table 6, Table 7 and Table 8, respectively. Noteworthy, in the CRNN model, we utilize a configuration comprising eight convolutional layers, five maxpooling layers, two batch normalization layers, and two bidirectional gated recurrent unit (GRU) layers. The convolutional layers employ a 3 × 3 kernel size, except for the final layer which uses a 2 × 2 kernel size. The number of filters in the convolutional layers starts at 64, increases to 128, and reaches 512. All maxpooling layers are 2 × 2 in size. Each of the bidirectional GRU layers contains 128 units. The input CAPTCHA images are transformed into grayscale and resized to 64 × 256 dimensions. The convolutional and maxpooling layers are parameterized to generate seven 512-dimensional feature sequences, which are subsequently fed into the GRU layers. To train this CRNN model, we employ the connectionist temporal classifier (CTC) loss function [40]. The comparison is conducted on the testing set of each CAPTCHA scheme with respect to the total character accuracy, overall CAPTCHA accuracy, non-trainable and trainable parameters, and size of weights on the hard disk. For the BoC CAPTCHA scheme results in Table 5, it is clear that the CRNGS_4s is the highest in terms of the total character accuracy, while the CRNN model is the best with regard to the overall CAPTCHA accuracy. On the other hand, for the Weibo CAPTCHA scheme achieved scores, it is obvious that the GRNGS_1s model surpasses all the other models in terms of both the total character accuracy and the overall CAPTCHA accuracy. For the Captcha 0.3 scheme, the attained results manifest that the CRNGS_4s exceeds the other models with regard to both total character accuracy and overall CAPTCHA accuracy. While for the Gregwar CAPTCHA scheme, the CRNGS_1s outperforms all the other model in both total character accuracy and overall CAPTCHA accuracy.

It can be noticed from the obtained score for all CAPTCHA schemes and all models that, for each CAPTCHA scheme, there is one of the proposed model versions that best fits this CAPTCHA scheme and achieved the best performance. These findings demonstrate that our proposed method is more generic and attains a high performance. For any CAPTCHA scheme, we can build several versions of our model with different number of softmax layers and train these model versions on the CAPTCHA scheme, and then select the model version with the highest accuracy as the final model. In our proposed approach, the number of softmax layers is converted into a hyperparameter that can be adjusted to fit the CAPTCHA scheme. For the storage size and the number of parameters evaluation metrics, it can be observed from the attained results that the CRNGS_1S is the lowest in size and number of trainable parameters. Additionally, as the number of softmax layers increased, both the storage size of the weights and the number of trainable and non-trainable parameters are also increased. It can also be observed that the CRNN model is the largest in terms of storage size and number of parameters.

## 5. Discussion

This paper introduced a CAPTCHA recognition method and procedure that is both flexible and efficient. This proposed algorithm can be analyzed from multiple perspectives, including efficiency, storage size, flexibility, and training time. Both advantages and drawbacks of the proposed approach will be covered in this section.

Regarding the efficiency, the proposed models manifest an excellent performance and relatively high accuracy in breaking the CAPTCHA resistance mechanisms successfully and extracting the CAPTCHA characters. These resistance mechanisms include character overlapping, noise lines, rotation, distortion, color background, and availability of multiple CAPTCHA character categories. According to the obtained results, the Gregwar CAPTCHA scheme demonstrated the strongest resistance mechanisms among the adopter schemes, and our proposed frameworks showed excellent ability in penetrating the resistance mechanisms of the adopted schemes and distinguishing the CAPTCHA characters successfully. Additionally, by introducing the concept of ABIs, the need for segmenting the CAPTCHA image into individual characters have been avoided, leading to faster recognition time and higher accuracy.

Regarding the flexibility, as can be observed from the proposed models, by selecting a different number of softmax layers (which represents the group size) a different number of ABIs is also set, and a model with a new architecture is built. Selecting a different number of softmax layers provides us with a range of models that can be used to recognize the CAPTCHA image, and the model with the highest accuracy rate will be selected as the best performance model.

In our proposed CAPTCHA recognition model, we have assumed that the CAPTCHA schemes used to train and test the proposed approach have a fixed number of characters, i.e., the images of a CAPTCHA scheme contain a fixed number of characters. In our approach, the number of characters *k* of a CAPTCHA scheme need to be determined at first, then the remaining hyperparameters including the number of softmax layers *t* and the number of ABIs *n* can be selected accordingly. However, the proposed approach can also be utilized with variable length CAPTCHA schemes. First, the maximum length of CAPTCHA characters needs to be specified, and then, the group size and number of groups can easily be selected to build the models. Second, an extra character (the empty character) needs to be added to the categories of the characters of a CAPICHA scheme for representing the blank characters when the length of the CAPTCHA’s characters is less than the maximum length.

The utilization of ABI brings several noteworthy advantages. It eliminates the need for segmenting CAPTCHA images into individual characters, enabling the model to locate and recognize characters simultaneously, and avoiding segmentation-related inefficiencies. Additionally, the model simplifies its architecture with the number of neurons in the softmax layer, regardless of the number of characters in the CAPTCHA, resulting in reduced storage requirements and parameters. Regarding the storage size, in our propose approach, as the group size increases (the number of the softmax layers increases), the models’ size and the number of trainable parameters also amplifies, leading to additional storage size in the hard disk. On the other hand, increasing the group size leads to a reduction in the number of ABIs for the model, and consequently decreasing the time needed to train and test the models. It is clear that the relationship between the storage size and time is a tradeoff, while the accuracies of the different versions of the model are almost similar.

However, the primary limitation of this work is the significant increase in the training and testing time due to the expansion of the training and testing sets, which is necessary for the CRNGS algorithm. This expansion occurs because *n* copies of each input CAPTCHA image are created for the datasets. This results in a proportional increase in both training and testing times. However, it is noted that recent hardware resources have become more powerful and can mitigate this issue.

## 6. Conclusions

This study introduces a novel algorithm for recognizing CAPTCHA images without the need for segmentation. The algorithm utilizes deep learning techniques and ABI character grouping, which involves making multiple copies of the CAPTCHA images to recognize and locate the characters. By incorporating ABI character grouping into the system, the recognition system’s overall size is reduced. In addition, the CNN model structure’s complexity is decreased since the output softmax layer’s neuron count remains fixed regardless of how many characters are in the CAPTCHA image. Additionally, by avoiding the segmentation step through the use of ABI character grouping, adopting a powerful feature extraction CNN architecture, and employing CRNGS for character localization, the proposed algorithm significantly improves CAPTCHA character recognition accuracy. The algorithm is evaluated on four different datasets, each containing approximately 70,000 images. Based on the experimental results, we demonstrate that our algorithm achieves higher CAPTCHA recognition accuracy than state-of-the-art models across the four CAPTCHA schemes. Moreover, the proposed model has a much smaller storage size than the compared models.

## Figures and Tables

**Table 1:** Accuracies of individual characters and overall CAPTCHAs using BoC CAPTCHA scheme.

	BoC CAPTCHA Scheme			
	**CRABI (CRNGS_1s)**	**CRNGS_2s**	**CRNGS_3s**	**Multi-Label (CRNGS_4s)**
1st Character Accuracy	98.78% (9878/10,000)	98.61% (9861/10,000)	98.49% (9849/10,000)	98.92% (9892/10,000)
2nd Character Accuracy	98.73% (9873/10,000)	98.57% (9857/10,000)	98.96% (9896/10,000)	99.02% (9902/10,000)
3rd Character Accuracy	97.72% (9772/10,000)	99.09% (9909/10,000)	99.01% (9901/10,000)	99.88% (9988/10,000)
4th Character Accuracy	98.56% (9856/10,000)	98.86% (9886/10,000)	99.06% (9906/10,000)	99.12% (9912/10,000)
Total Character Accuracy	98.44% (39,379/40,000)	98.78% (39,513/40,000)	97.68% (39,552/40,000)	99.03% (39,614/40,000)
Overall CAPTCHA Accuracy	94.33% (9433/10,000)	95.41% (9541/10,000)	95.82% (9582/10,000)	96.39% (9639/10,000)

**Table 2:** Accuracies of individual characters and overall CAPTCHAs using Weibo CAPTCHA scheme.

	Webo CAPTCHA Scheme			
	**CRABI (CRNGS_1) [35]**	**CRNGS_2s**	**CRNGS_3s**	**Multi-Label (CRNGS_4s)**
1st Character Accuracy	98.70% (9870/10,000)	98.01% (9801/10,000))	82.81% (8281/10,000)	98.26% (9826/10,000)
2nd Character Accuracy	98.35% (9835/10,000)	94.00% (9400/10,000)	81.00% (8100/10,000)	94.36% (9436/10,000)
3rd Character Accuracy	95.83% (9583/10,000)	96.65% (9821/10,000)	80.13% (8013/10,000)	93.85% (8281/10,000)
4th Character Accuracy	98.68% (9868/10,000)	98.21% (9821/10,000)	83.98% (8398/10,000)	97.64% (9764/10,000)
Total Character Accuracy	97.89% (39,156/40,000)	96.72% (38,687/40,000)	81.98% (32,792/40,000)	96.02% (38,411/40,000)
Overall CAPTCHA Accuracy	92.68% (9268/10,000)	88.75% (8875/10,000)	85.93% (8593/10,000)	86.24% (8624/10,000)

**Table 3:** Accuracies of individual characters and overall CAPTCHAs using Captcha 0.3 CAPTCHA scheme.

	Captcha 0.3 CAPTCHA Scheme			
	**CRABI (CRNGS_1s)**	**CRNGS_2s**	**CRNGS_3s**	**Multi-Label (CRNGS_4s)**
1st Character Accuracy	99.92% (49,958/50,000)	98.49% (9849/10,000)	98.81% (9881/10,000)	99.46% (9946/10,000)
2nd Character Accuracy	99.90% (49,951/50,000)	95.26% (9526/10,000)	96.87% (9687/10,000)	98.16% (9816/10,000)
3rd Character Accuracy	98.66% (49,331/50,000)	95.70% (9570/10,000)	96.15% (9615/10,000)	97.91% (9791/10,000)
4th Character Accuracy	99.28% (49,642/50,000)	98.39% (9839/10,000)	98.89% (98.89/10,000)	99.32% (9932/10,000)
Total Character Accuracy	96.11% (38,444/40,000)	96.96% (38,784/40,000)	97.68% (39,072/40,000)	98.71% (39,485/40,000)
Overall CAPTCHA Accuracy	85.93% (8593/10,000)	89.16% (8916/10,000)	91.62% (9162/10,000)	95.33% (9533/10,000)

**Table 4:** Accuracies of individual characters and overall CAPTCHAs using Gregwar CAPTCHA scheme.

	Gregwar CAPTCHA Scheme			
	**CRABI (CRNGS_1s) [35]**	**CRNGS_2s**	**CRNGS_3s**	**Multi-Label (CRNGS_4s)**
1st Character Accuracy	93.12% (9312/10,000)	88.83% (8883/10,000)	87.05% (8705/10,000)	88.69% (8869/10,000)
2nd Character Accuracy	85.28% (8528/10,000)	74.17% (7417/10,000)	79.59% (7959/10,000)	78.43% (7843/10,000)
3rd Character Accuracy	74.03% (7403/10,000)	80.65% (8065/10,000)	78.70% (7870/10,000)	78.17% (7817/10,000)
4th Character Accuracy	88.68% (8868/10,000)	88.37% (8837/10,000)	87.79% (8779/10,000)	87.93% (8793/10,000)
Total Character Accuracy	85.28% (34,111/40,000)	83.00% (33,202/40,000)	83.28% (33,313/40,000)	83.30% (33,322/40,000)
Overall CAPTCHA Accuracy	54.20% (5420/10,000)	49.78% (4978/10,000)	50.77% (5077/10,000)	51.23% (5123/10,000)

**Table 5:** Comparison Results Using BoC CAPTCHA Scheme.

	BoC CAPTCHA Scheme				
	**CRABI** **(CRNGS_1s)**	**Multilabel** **(CRNGS_4s)**	**CRNN**	**CRNGS_2s**	**CRNGS_3s**
Testing Total Character Accuracy	98.44% (39,379/40,000)	99.03% (39,614/40,000)	-	98.78% (39,513/40,000)	97.68% (39,552/40,000)
Testing Overall CAPTCHA Accuracy	94.33% (9433/10,000)	96.39% (9639/10,000)	96.47% (9647/10,000)	95.41% (9541/10,000)	95.82% (9582/10,000)
Non-trainable and Trainable Parameters	6,640,090	7,838,248	10,478,875	7,039,476	7,484,945
Size of Weights on Hard Disk	79.9 MB	94.1 MB	125.1 MB	84.7 MB	90.0 MB

**Table 6:** Comparison Results Using Weibo CAPTCHA Scheme.

	Weibo CAPTCHA Scheme				
	**CRABI** **(CRNGS_1s) [35]**	**Multilabel** **(CRNGS_4s) [35]**	**CRNN [35]**	**CRNGS_2s**	**CRNGS_3s**
Testing Total Character Accuracy	97.89% (39,156/40,000)	96.03% (38,411/40,000)	-	96.72% (38,687/40,000)	81.98% (32,792/40,000)
Testing Overall CAPTCHA Accuracy	92.68% (9268/10,000)	86.24% (8624/10,000)	91.05% (9105/10,000)	88.75% (8875/10,000)	85.93% (8593/10,000)
Non-trainable and Trainable Parameters	6,670,812	7,961,136	10,477,853	7,100,920	7,577,111
Size of Weights on Hard Disk	25.5 MB	30.4 MB	40 MB	28.5 MB	28.9 MB

**Table 7:** Comparison Results Using Captcha 0.3 CAPTCHA Scheme.

	Captcha 0.3 CAPTCHA Scheme				
	**CRABI** **(CRNGS_1s)**	**Multilabel** **(CRNGS_4s)**	**CRNN**	**CRNGS_2s**	**CRNGS_3s**
Testing Total Character Accuracy	96.11% (38,444/40,000)	98.71% (39,485/40,000)	-	96.96% (38,784/40,000)	97.68% (39,072/40,000)
Testing Overall CAPTCHA Accuracy	85.93% (8593/10,000)	95.33% (9533/10,000)	83.57% (8357/10,000)	89.16% (8916/10,000)	91.62% (9162/10,000)
Non-trainable and Trainable Parameters	7,193,086	10,050,232	10,486,591	8,145,468	9,143,933
Size of Weights on Hard Disk	27.5 MB	38.4 MB	40.1 MB	31.8 MB	34.9 MB

**Table 8:** Comparison Results Using Gregwar CAPTCHA Scheme.

	Gregwar CAPTCHA Scheme				
	**CRABI** **(CRNGS_1s) [35]**	**Multilabel** (**CRNGS_4s) [35]**	**CRNN [35]**	**CRNGS_2s**	**CRNGS_3s**
Testing Total Character Accuracy	85.28% (34,111/40,000)	83.31% (33,322/40,000)	-	83.00% (33,202/40,000)	83.28% (33,313/40,000)
Testing Overall CAPTCHA Accuracy	54.20% (5420/10,000)	51.23% (5123/10,000)	49.98% (4998/10,000)	49.78% (4978/10,000)	50.77% (5077/10,000)
Non-trainable and Trainable Parameters	7,193,086	10,050,232	10,486,591	8,145,468	9,143,933
Size of Weights on Hard Disk	27.5 MB	38.4 MB	40.1 MB	32.7 MB	34.9 MB

## Data Availability

Data are contained within the article.
